# Communication access, public health information sources, and language preference during the COVID-19 pandemic in Indigenous communities in Northwest Territories, Canada

**DOI:** 10.1371/journal.pone.0330394

**Published:** 2025-11-18

**Authors:** Rachel Harris, Fariba Kolahdooz, Afsaneh Omidimorad, Adrian Wagg, Debbie DeLancey, Kami Kandola, Sami Pirkola, Stephanie Irlbacher-Fox, André Corriveau, Sangita Sharma

**Affiliations:** 1 Indigenous and Global Health Research Group, Department of Medicine, Faculty of Medicine & Dentistry, College of Health Sciences, University of Alberta, Edmonton, Alberta, Canada; 2 Division of Geriatric Medicine, Department of Medicine, Faculty of Medicine & Dentistry, College of Health Sciences, University of Alberta, Edmonton, Alberta, Canada; 3 Aurora College, North Slave Campus, Yellowknife, Northwest Territories, Canada; 4 Department of Health and Social Services, Government of the Northwest Territories, Yellowknife, Northwest Territories, Canada; 5 Faculty of Social Sciences, Tampere University, Tampere, Finland; 6 Hotıì ts’eeda Northwest Territories SPOR Support Unit, Yellowknife, Northwest Territories, Canada; 7 Independent public health consultant for Northwest Territories and Nunavut, Yellowknife, Northwest Territories, Canada; Institute of Tropical Medicine / University of Antwerp, BELGIUM

## Abstract

Effective communication during a public health emergency, such as the COVID-19 pandemic, has been identified by the World Health Organization as a challenge; it is also critical to ensuring more positive public health outcomes. As such, given the already limited health resources available in remote communities, this project aimed to explore communication access, sources of COVID-19 information, and language preference among Indigenous communities in Northwest Territories, Canada.

Data collection occurred between April-November 2021 in ten Northwest Territories communities. Utilizing convenience sampling methods due to public health restrictions, self-identifying Indigenous adults (≥18 years) were invited to complete a semi-structured interviewer administered questionnaire. During interviews, relevant public health measures were upheld (i.e., wearing masks and maintaining distance). Open-ended questions regarding communication access, sources of COVID-19 information, and language preference were asked.

**Results:**

Participants (n = 287; 31.1% male; mean age 41.6 years, SD = 13.5) reported accessing three main forms of communication: radio (84.7%), the internet (80.5%), and cable television (71.8%). Some participants (19.5%) reported having no internet access at home. Participants’ main sources of COVID-19 information were websites (35.3%), social media (26.5%), and the news delivered via television or newspaper (48.4%). Only 11.0% of participants acquired information from healthcare workers. Some participants (9.9%) preferred to receive information in the traditional languages of the Dene (Dene Ked’e, Tłıchǫ) and Anishinaabe.

Ensuring communication access by providing adequate internet access in all communities and producing information sources in preferred languages should be a priority. These results can inform future public health policy for Northwest Territories.

## Introduction

The World Health Organization (WHO) announced on March 11, 2020, that the Coronavirus disease 2019 (COVID-19) outbreak had become a pandemic. Communication during such a public health emergency has been identified by the WHO as one of the biggest challenges to the adoption of mitigation strategies [1]. Effective communication during a health crisis is critical to saving lives [2] and being well-informed during an infectious disease outbreak enables governments and the public to understand and manage the health crisis [3]. For instance, success in Japan regarding minimising the effects of COVID-19 has been attributed to the effective communication of public health messages [4]. As information received at the community level is imperative to controlling an infectious disease outbreak, there is a heightened need to understand how the public gain access to health information and in what form. Understanding communication access in the home and sources of information is crucial, as these serve as precursors to COVID-19 preventative behaviors.

The Government of Northwest Territories (GNWT), which governs and manages public services in Northwest Territories (NWT), including healthcare, declared COVID-19 to be a public health emergency on the 18^th^ of March 2020. The forested landscape and tundra of NWT, one of three Northern territories in Canada, extends for more than 1,171,918 km^2^ and is home to an estimated 41,070 individuals [5]. Many individuals in NWT, 49.6% of whom are Indigenous [6], live in remote communities and experience challenges when accessing healthcare [7]. Effective dissemination of evidence-based public health information, which helps to mitigate the transmission of COVID-19 through use of public health measures, is particularly important in such remote communities with limited health resources.

During past infectious disease outbreaks, such as the 2003 SARS outbreak in the Netherlands, mainstream media sources such as television or newspapers were the predominant sources of information [8]. Hard copies of the territorial and regional newspapers are available weekly in NWT, however, in remote and isolated communities this may not be the case. Furthermore, over recent years there has been a shift from relying on mainstream news outlets toward utilizing other sources of information, including social media [9]. During the COVID-19 pandemic, mass media (television and radio), print media (newspapers and posters), and the internet have been used worldwide to communicate precautionary and supportive measures [10]. A survey from 2020 reported the internet (77.1%) as the most common source of COVID-19 information, including social media such as Facebook [11]. However, despite the accessibility of social media, there are concerns that the content is generally unverified [12] and updated frequently from varied sources which introduces a higher level of inconsistency [13] and may also be a source of misinformation [14].

There is limited literature available regarding sources of COVID-19 information, and language preferences. The project collected data on the broader impacts of COVID-19 and explored home communication access, sources of COVID-19 information, and language preferences in ten NWT communities. The aim of this paper was to address the gap in knowledge, to inform public health communications initiatives, including the development of innovative approaches to disseminating effective and reliable public health measures and guidelines in Indigenous communities in NWT.

## Materials and methods

### Project design and setting

The project utilized a convergent, mixed-methods cross-sectional design and took place within ten NWT communities, which varied in remoteness, size, and infrastructure. Three communities were larger (>1000 people), three communities were medium-sized (300–1000 people), and four communities were small (<300 people). Further details have been published elsewhere [15]. The large and medium-sized communities have year-round road access and community health centres. The small communities are without year-round road access, and only some have health centres.

In all aspects of the project, the community-based participatory research (CBPR) model was utilized [16], which ensured the participation of Indigenous communities as equal partners at all stages of the research process, helping to build trusting relationships between the community members and researchers.

### Project population and recruitment

All participants in this project were self-identifying Indigenous adults, ≥ 18 years of age, and had resided in one of the communities for two months prior to the start of data collection. In each community, local research assistants were trained to recruit and interview participants. Due to public health restrictions at the time, community-specific convenience recruitment methods were utilized. These included the direct calling of community members and organizations, social media posts on Facebook, word of mouth, and passive advertising in various community locations.

### Data collection

The study design enabled the concurrent collection and analysis of quantitative and qualitative data, providing a thorough understanding of the research question. Between April and November 2021, quantitative and qualitative data were collected via a semi-structured questionnaire which included both closed- and open-ended questions. The questionnaire provided a framework with pre-defined questions to ensure consistency across participants, while also allowing participants to provide more detailed information. The questionnaire was administered by trained research assistants in the local Indigenous language or English. Participants were asked two questions regarding communication access and sources of information: “Which of the following do you have regular access to at home? 1. Internet, 2. Cable TV, 3. Local TV, 4. Radio, 5. Newspaper, 6. None of the above”; and “Where do you get information about COVID-19 and COVID-19 prevention?”. With regard to language preference, the following questions were asked: “Did you receive COVID-19 health and prevention (including vaccine) information in the language you prefer?”; and “If not, what language would you like to receive COVID-19 health and prevention information in?”. For all questions, participants were given the opportunity to add any further information, if desired.

Each participant gave verbal informed consent before the start of the interview. Interviews were conducted by phone or face-to-face, and were audio-recorded with the participant’s permission. Face-to-face interviews were conducted at a time and location convenient to the participant. Public health measures were upheld with the wearing of masks and social distancing of at least 6 feet being maintained during face-to-face interviews. Responses to open-ended questions were transcribed verbatim and verified by checking the transcribed data against the audio files. Unique identification numbers linked each participant’s anonymous data entry into an electronic case report form using REDCap (version 8.1.1). Each participant received a $50 gift card honorarium.

### Ethical considerations

Ethical approval was granted by the University of Alberta’s Research Ethics Board and a research license was obtained from the Aurora Research Institute in Inuvik, NWT. Formal research agreements and memorandums of understanding were signed by all stakeholders before data collection commenced. A Community Advisory Board (CAB), composed of Elders, local healthcare professionals, community members, key government officials, and policymakers, provided guidance in all aspects of this project and ensured that questions were culturally appropriate. In collaboration with Indigenous partners and the CAB, a collective decision was made to exclude participant IDs from the manuscript. This decision reflects a commitment to cultural sensitivity and honors the lived experiences of Indigenous community members. Historically, Indigenous individuals were often referred to by numbers rather than names in institutional systems, a dehumanizing practice tied to Canada’s colonial legacy and the resulting intergenerational trauma. Details such as gender or age were also not reported, as some participating communities have fewer than 300 members. Including such descriptive information could unintentionally make individuals identifiable through the quotations. Additional information regarding the ethical, cultural, and scientific considerations specific to inclusivity in global research is included in the Supporting Information (S1 File)”

### Data analysis

Quantitative data analyses were performed utilizing SAS statistical software, (SAS Version 9.4, SAS Institute Inc., Cary, NC). The frequencies of communication modes and sources of COVID-19 information utilized by participants were expressed as proportions (%). Ages were classified into four age groups in years (18–29, 30–49, 50–65, and > 65). Educational levels were classified into post-secondary (vocational or skills training, and university) or high school and below. The Fisher’s Exact Test was utilized to determine if there was a significant association between gender, age group (older ≥ 50 years or younger 18–49 years), educational level, and community size, and both home communication access and preferred language. A two-tailed *P* value of <0.05 indicated statistical significance.

Qualitative data were analyzed using deductive thematic analysis. After familiarization with the qualitative data, quotes were coded and categorized into themes which were identified through several rounds of reading and coding. Two analysts performed the coding independently, and worked with another team member to resolve disagreements. The combination of the methods supported a robust analysis by merging statistical data with personal narratives.

## Results

### Sociodemographic characteristics

287 community members (mean age = 41.6 years; SD ± 13.5; 33.1% men) participated. Of the interviews, 197 were conducted over the phone, and 90 were conducted face-to-face. The mean interview length was 45 minutes. Most participants (73.5%) were between 18 and 49 years old, 5.7% were > 65 years, and the majority (59.9%) lived in large communities. The majority of participants (77.7%) had completed high school education or above. The most common self-identified ancestry was First Nations (72.5%), followed by Métis (21.6%) and Inuit (4.5%) (Table 1).

**Table 1:** Sociodemographic characteristics of participating community members in ten Northwest Territories communities, Canada (n = 287).

Characteristics	Frequency (%)
**Gender identity**	
Man	95 (33.1)
Woman	191 (66.6)
Refused	1 (0.3)
**Age in years (mean ± SD)**	41.6 (± 13.5)
^**a**^**Age group (years)**	
18-29	58 (20.5)
30-49	150 (53.0)
50-65	59 (20.8)
> 65	16 (5.7)
^**b**^**Community size (# people)**	
**Large (>1000)**	169 (59.9)
**Medium (300–1000)**	79 (28.0)
**Small (<300)**	34 (12.1)
**Educational status**	
^§^ Post-secondary	88 (30.7)
High school diploma or higher	135 (47.0)
Junior high school	43 (15.0)
Elementary school	11 (3.8)
Other (none/unsure/declined)	10 (3.5)
**Ethnicity**	
First Nations	208 (72.5)
Metis	62 (21.6)
Inuit	13 (4.5)
^**c**^ Other	4 (1.4)

SD (standard deviation); ^**a**^ Missing =4; ^**b**^ Missing = 5; ^**c**^ Other = not specified

§Post secondary: includes vocational or cultural training, and university education

### Home communication access

Community members most frequently reported having access to the radio (84.7%), the internet (80.5%), and cable television (71.8%) in the home; half of the participants had access to local television stations (50.2%) and newspapers (47.0%). A small proportion (2.1%) of participants did not have home communication access via radio, newspaper, television, or the internet (Table 2), and 19.5% of homes did not have internet access. Of the total sample, participants 18–29 years old (n = 58) most frequently reported having home communication access to the internet (17.3%) and radio (16.6%), with newspapers (8.1%) being the least reported. Older community members (>50 years old; n = 75) most frequently reported having home communication access to the radio (22.6%), cable television (21.2%), and the internet (20.1%), with newspapers (13.4%) being least reported.

**Table 2:** Regular home communication access and sources of COVID-19 public health information used among participating community members in ten Northwest Territories communities, Canada.

Communication access* (n = 287)	Frequency (%)
Radio	243 (84.7)
Internet	231 (80.5)
Cable television	206 (71.8)
Local television	144 (50.2)
Newspaper	135 (47.0)
None of the above	6 (2.1)
Source of information * (n = 283)	**Frequency (%)**
News (television or newspaper)	137(48.4)
Website	100 (35.3)
Social media	75 (26.5)
Government	54 (19.1)
Radio	43 (15.2)
Healthcare worker	31 (11.0)
Workplace	20 (7.1)
Word of mouth	18 (6.4)

(*Percentage addition might be > 100 as more than one answer could be selected)

### Sources for COVID-19-related information

Participants most frequently utilized three sources of COVID-19 information: the news delivered via television or newspaper (48.4%), websites (35.3%), and social media (26.5%). Healthcare workers and other individuals (coworkers, friends, and family members) were a source of information for 11.0% of participants (Table 2). Some participants (9.9%) preferred to receive information in the Dene (Dene Ked’e, Tłıchǫ) languages.

Statistically significant associations were identified between educational level and internet access (p = 0.003), with participants having a high school education or lower being more likely to have internet access. Additionally, significant associations were found between age and cable television access (p = 0.027) as well as local television access (p = 0.002), with younger participants (aged 18–49years) being more likely to have access to cable and local television.

(Table 3). There were also statistically significant associations found between large community size and having access to local television (p < 0.001), cable television (p = 0.002), the internet (p = 0.001), and information in the preferred language (p = 0.004). Females were more likely than males to have access to cable TV (p < 0.001).

**Table 3:** Regular home communication access by age group, gender identity, community size and educational level among participating community members in ten Northwest Territories communities, Canada (n = 277).

	Internet (%)	Cable television (%)	Local television (%)	Radio (%)	Newspaper (%)
**Age group (years)**					
18-49	16.4	51.6	33.9	63.2	34.7
≥ 50	19.1	19.9	16.3	21.3	13.4
*p-value	0.091	**0.027**	**0.002**	0.149	0.062
**Gender identity**					
Man	25.1	19.2	15.7	27.5	17.8
Woman	55.1	52.6	34.5	56.8	29.3
*p-value	0.360	**<0.001**	0.491	0.770	0.132
**Community size**					
Large	52.1	47.2	35.8	51.1	27.0
Medium	20.9	17.7	11.0	24.5	14.5
Small	7.5	6.4	3.2	8.9	5.3
*p-value	**0.001**	**0.002**	**<0.001**	0.164	0.576
**Educational level**					
High school or below	51.6	48.4	35.4	57.8	32.5
Vocational/skills training and university	28.5	23.5	15.2	26.7	14.1
*p-value	**0.003**	0.101	0.084	0.139	0.090

*Fisher’s Exact Test: statistically significant p-values are in bold type

### Qualitative results: Sources of information

In addition to utilizing the WHO and the Centres for Disease Control and Prevention websites, participants reported that the GNWT website provided comprehensive, up-to-date, and reliable information on case numbers, travel protocols, and isolation requirements, which were specific to the region of NWT. Searching within the GNWT website was also described by participants as user-friendly:

*“Actually, the NWT has a great site and they have all the info listed and they add daily new info so it’s pretty up to date and pretty straightforward, and has everything if you are going to be travelling and your isolation plan and your forms. Everything is in that one area, so it’s very user-friendly too, and you can ask it questions too and put in a search and it will bring up the information for you, if it doesn’t have the info it gives you contact numbers of where to get the information. And they have news releases and press releases too when there is a COVID case”.*

*“From the GNWT dashboard. They have a COVID-19 specific dashboard which has all of the numbers and cases and for...that’s for the NWT specific”.*

*“COVID19 info dashboard shows all the people being vaccinated, what percentage, where COVID is in the territories, if there are non-residents, yeah it’s on the internet”.*

Participants also reported obtaining COVID-19 information through the news, primarily via the Canadian Broadcasting Corporation (CBC), on cable and local television, the radio, and in the newspaper. Participants explained that television provided COVID-19 updates throughout the day and that these sources provided both local and national COVID-19 information. Participants also described moving from one source to another for COVID-19 updates:

*“I got the information from the news. We always watch news like what happens in the world. We always watch the news like every day, because you never know what’s going to happen”.*

*“All sorts of news but the biggest thing I have been paying attention to is CBC news and anything that has been affiliated with the North”.*

*“And then through CBC for national news about the virus”.*

*“Our territorial newspapers”.*

*“CBC radio and also postings in the local like the post office and the stores that you go into, they are all posting, you cannot miss any of those information”.*

*“When I go to work and I get the emails, and my husband reads CBC North and will keep me updated when a post comes up”.*

Participants found mobile apps, such as the COVID alert app and Snapchat, to be a convenient and fast way to access reliable and up-to-date COVID-19 information. Many participants specifically discussed utilizing Facebook as an information source and as a way to access government updates:

*“uh a lot on the government website on Facebook”*

*“I see a lot on my twitter, and Facebook and snap. Like every time I log on to Facebook it says. There is even COVID rules on Facebook”.*

*“I know that probably not everyone was using it, but I felt like it gave that COVID alert app. Staying up to date when the chief public health officers of each of where we are, whenever they were doing updates, it was like let’s tune in, see what’s happening, we’d get a little more information”.*

However, some participants questioned the credibility of information on Facebook:

*“We try to watch where our information is coming from and read internet pages rather than other’s Facebook statuses”.*

Participants also noted that the radio was a source for information from health professionals, such as nurses working in local health centres. Handouts obtained from the hospital or health centres were also mentioned as a source of information:

*“the health staff was on the local radio”.*

*“on local radio the health centre they go on the radio and provide information”.*

Some participants explained that workplaces provided information and updates via email or phone to staff:

*“Online, we got most of it online and in emails and my workplace provided an update on a regular basis to send a message out and awareness of COVID-19 and prevention”.*

*“through my work we get 3 times a week we get brief notes from the government where I work so that is through my work phone”.*

For some participants, family and friends were the only means of communication in NWT communities:

*“well friends, family, that’s the only way I know anything about the world”.*

### Language preferences

Participants most frequently reported English as the preferred language for COVID-19 information. Some participants also mentioned information being available in both English and Indigenous traditional languages:

*“Yeah, in our language and in English”.*

*“They have a booklet in our language and English, so they teach both languages, so people will understand”.*

*“Here it was translated to* Tłıchǫ, *so yes”.*

Quantitative and qualitative data enabled an extensive understanding of communication access, public health information sources, and language preference during the COVID-19 pandemic in Indigenous communities in NWT. Quantitative data revealed measurable communication access, sources of information and language preferences, while qualitative data offered deeper insights through narratives.

## Discussion

The project provides a timely assessment of communication access and COVID-19 information sources in Indigenous communities within NWT. Participants accessed various sources of COVID-19 information including the broadcast news, websites, and social media, and expressed a preference for information to be available in both English and the local traditional language. A reliance on radio, the internet, and cable television to access communication in the home was identified, with younger participants (18–29 years old) most frequently utilizing the internet and radio. This reliance has been previously described in other settings [17].

One of the main sources of COVID-19 information accessed by participants was the mainstream news delivered via television or newspapers; few community members received information from healthcare workers, the workplace, or family and friends. Studies from past outbreaks of infectious diseases, including severe acute respiratory syndrome (SARS) [8], the influenza A virus subtype H1N1 [18], and seasonal flus [19], have also identified the popularity and importance of the television and newspapers for information, despite these sources being perceived as relatively untrustworthy [9].

Globally, during the early stages of the pandemic when isolation and social distancing were recommended, the internet provided readily accessible information on COVID-19 [20]. This was reflected among many participants in this project who received COVID-19 information through online websites. The official website of the GNWT was the main source of comprehensive COVID-19 information. The GNWT website promoted the use of an 811 number as a source of public communication through which to reach the established regulatory, self-isolation, and regional centre accommodations lines [21]. Health information of varying quality is accessible online, and social media platforms often fail to differentiate between credible and non-credible sources, leaving individuals to assess reliability without aid. Previous research has identified factors such as personal beliefs, social circumstances, and health literacy as influencing an individual’s judgment of a source’s trustworthiness [22]. In the United States, such government sources for COVID-19 information were highly trusted among the public [23]. As such, participants in this project reporting being without home internet access is of concern. Despite the Canadian government recognizing internet access as an essential service in 2016 [24], affordable, reliable, and accessible internet services in remote Indigenous communities remains limited [25].

Participants also relied on social media, including Facebook and Snapchat, for COVID-19 information, reflecting a current trend in global communications [26]. Web-based apps, such as the ‘COVID alert app’ reported in this project, have made information regarding COVID-19 more easily accessible [27]. However, caution is warranted as the content on such online platforms is unregulated and not subject to peer review [12], and misinformation spread on social media may be detrimental to public health [28]. Further, the negative effects of social media, such as information overload, may alter personal coping strategies related to COVID-19 [29] and result in adverse psychological outcomes [3].

Future efforts to disseminate evidence-based information during public health emergencies, such as COVID-19 and other infectious disease outbreaks, should account for the diverse communication preferences of community members. Within public health communication strategies, utilizing population-based demographics (age and sex) may help with generating information in relevant formats, languages, tones, and media. The development of culturally appropriate web-based information, developed in collaboration with local Indigenous community members, should be pursued. Providing government subsidies for internet services, increasing the availability of free apps, and increasing the visibility of reliable information on search engines should also be considered. Leveraging healthcare workers as a source of evidence-based information and ensuring that public health messaging in available in both English and local languages is encouraged.

This project has some limitations due to COVID-19 public health restrictions at the time of data collection, participant recruitment utilizing convenience sampling methods, and 66.6% of participants being female. The potential underestimation of the preference for Dene languages is recognized; as noted, the 9.9% figure reflects only participants who reported not receiving information in a preferred language and then specified Dene as the preferred language. This approach does not capture participants who received information in Dene and were therefore not prompted to report a language preference. This may have led to an underestimation of the true demand for Dene-language materials. Only 50.7% of individuals in NWT are female, which may have introduced bias. The over-representation of participants from larger communities (59%) and the under-representation of participants aged 65 + years (16%) may also be sources of bias, particularily regarding the capture of preferred languages. These factors potentially limit the generalizability of this project’s results. However, the recruitment of participants from ten communities which varied by ethnicity, size, and access to healthcare increases the representativeness of results. This study did not focus on disinformation, although exploring this topic would have been valuable. We recognise this as a limitation.

## Conclusions

This project provides a snapshot of a critical moment within a world event. For NWT communities, information on COVID-19 was mainly delivered via radio, television, newspapers, online websites, and social media. Future initiatives to disseminate evidence-based COVID-19 information should thoughtfully consider the varied communication preferences and language needs of community members. It is important to utilize multiple channels, such as television, radio, newspapers, websites, and web-based apps, to ensure effective outreach and accessibility for all. The project results can inform policymakers in Indigenous communities within NWT regarding effectively and reliably disseminating public health measures, guidelines, and information.

## Supporting information

S1 File Inclusivity-in-global-research-questionnaire_May 14. (DOCX)

S2 File GRAMM checklist_Soureces Info_29Apr2025. (DOCX)
