# Supplementary material for: Communication access, public health information sources, and language preference during the COVID-19 pandemic in Indigenous communities in Northwest Territories, Canada
Source: PLoS One. 2025 Nov 18;20(11):e0330394. doi: 10.1371/journal.pone.0330394 (PMC12626260; doi:10.1371/journal.pone.0330394)
Supplement: S2 File — (DOCX) [file pone.0330394.s002.docx]

| Guideline | Page information |
| --- | --- |
| 1. Describe the justification for using a mixed methods approach to the research question | Data collection pg.6 |
| 2. Describe the design in terms of the purpose, priority and sequence of methods | Materials and methods pgs. 5-8 |
| 3. Describe each method in terms of sampling, data collection and analysis | pgs. 5-7 |
| 4. Describe where integration has occurred, how it has occurred and who has participated in it | Data analysis pg. 7-8 |
| 5. Describe any limitation of one method associated with the present of the other method | Data analysis pg. 8 |
| 6. Describe any insights gained from mixing or integrating methods | Results pg.17 |

**Supplementary File 1: Good reporting of a mixed-methods study (GRAMMS) checklist**

Reference: O'Cathain A, Murphy E, Nicholl J. The quality of mixed methods studies in health services research. J Health Serv Res Policy. 2008;13: 92-98
